# Apathy in subarachnoid hemorrhage: study protocol for a 1-year follow-up study

**DOI:** 10.3389/fneur.2024.1358102

**Published:** 2024-07-31

**Authors:** Wai Kwong Tang, Kwok Chu George Wong

**Affiliations:** ^1^Department of Psychiatry, Chinese University of Hong Kong, Shatin, Hong Kong SAR, China; ^2^Department of Surgery, Chinese University of Hong Kong, Shatin, Hong Kong SAR, China

**Keywords:** apathy, subarachnoid hemorrhage, stroke, Functional Independence Measurement (FIM), Lawton Instrumental Activities of Daily Living Scale (IADL)

## Abstract

**Introduction:**

Apathy is a frequent and debilitating condition among subarachnoid hemorrhage (SAH) survivors. Few studies have evaluated apathy in SAH, and none have examined the course of the condition, predictors of persistent apathy, or its impact on functional outcomes. The proposed study will examine, for the first time, the 12-month course of apathy and its impact on functional outcomes in the largest cohort of SAH survivors to date.

**Methods and analysis:**

The current study is designed as a prospective cohort study with a duration of 36 months. We will recruit 240 participants. A trained research assistant will assess apathy using the Apathy Evaluation Scale 3 months after SAH. Patients’ level of functioning, comorbidity, global cognitive functioning, and depressive symptoms will be assessed. All SAH patients will participate in follow-up assessments of apathy and functioning at 9 (T2) and 15 months (T3) post-SAH or at 6 and 12 months after the first assessment. Predictors of persistent apathy and the impact of apathy on functional outcomes will be examined.

**Discussion:**

This will be the first large-scale 1-year follow-up study of apathy in SAH survivors. The findings will provide valuable data to advance our understanding of the clinical course of apathy in this population. Moreover, the results will have clinical relevance by providing essential information to patients, caregivers, and clinicians; promoting the evaluation of apathy; and facilitating the development of prevention strategies, rehabilitation programs, and therapeutic options.

**Ethics and dissemination:**

Ethical approval for this study was obtained from the Joint Chinese University of Hong Kong-New Territories East Cluster Clinical Research Ethics Committee (CREC Ref. No.: 2023.339) on 3 October 2023. The findings of this study will be shared through publication in a peer-reviewed journal, presentations at relevant conferences, and dissemination through social media platforms.

## Introduction

Subarachnoid hemorrhage (SAH) is a rare and severe type of stroke. It mainly affects individuals at a mean age of 55 years, potentially leading to the loss of many years of productive life. The rupture of an intracranial aneurysm is the underlying cause in 85% of SAH cases (1).

Estimates suggest that 55% of patients with SAH survive and regain independent function, whereas 19% remain dependent and 26% die (1). Many survivors experience long-term deficits in cognition, quality of life, mood, and fatigue (2). Neuropsychiatric conditions, such as apathy, depression, fatigue, anxiety, and posttraumatic stress disorder, are often neglected in these patients (3).

Apathy is broadly defined as a decrease in goal-directed behavior due to a loss of motivation (4), and is characterized by a general lack of emotion, interest, or concern (4). Patients with apathy exhibit a loss of motivation, concern, interest, and emotional responses. This manifests as decreases in initiative, interaction with the environment, and interest in socialization (5). Apathy is increasingly recognized as a syndrome. One set of criteria defines apathy as a loss of or reduction in motivation relative to the individual’s previous state, marked by decreased levels in at least 2 of the following three parameters: goal-directed behavior, cognitive activity, or emotion. Furthermore, the criteria stipulate that for a diagnosis of apathy, the symptoms must significantly impair daily functioning and cannot be attributed to physical or motor disabilities, decreased consciousness, or drug use (6). Although apathy can be a feature of depression, it is distinguishable from depressed mood in the context of neurological conditions, supporting the argument that these entities represent distinct constructs (7). Apathy in neurological diseases has been hypothesized to arise from defects in the frontal subcortical circuit, where the anterior cingulate circuit is specifically associated with motivation (5).

The clinical impact of apathy is becoming increasingly well-recognized. Apathy significantly contributes to poor outcomes in neurological populations, independent of depression. Moreover, apathy is associated with worsening social and functional impairments, decreased responsiveness to or compliance with treatment, poor awareness of behavioral and cognitive changes, and poor clinical outcomes and overall quality of life. Therefore, apathy imposes significant economic, social, and physical burdens. Patients with apathy experience greater distress and face earlier and more frequent institutionalization compared with patients with similar impairments but without apathy (8). Apathy is a common phenomenon associated with cerebral diseases, such as Alzheimer’s disease, Parkinson’s disease, traumatic brain injury, and stroke. Apathy is often undiagnosed and thus untreated (9), leading to delays in recovery, decreased social interaction, and increased caregiver burden (9). Apathy is the most frequent behavioral change observed in Alzheimer’s disease, affecting 19%–88% of patients (10). In these patients, apathy can accelerate functional declines, increase mortality (10), and add to caregiver distress (11). Similarly, apathy is observed in 25%–60% of patients with Parkinson’s disease (12), particularly those with cognitive impairments. Apathy is associated with reduced functioning in the activities of daily living, decreased treatment responses, poor outcomes, diminished quality of life, and emotional distress for caregivers (13). Moreover, apathy is a frequent consequence of head injuries, affecting up to 72% of patients (7). In this context, apathy is associated with unemployment (14), reduced quality of life, decreased participation in rehabilitation activities (7), poor psychosocial functioning (15), and a high caregiver burden (15). Previously, our group found that the prevalence of apathy in local stroke survivors was 10.8% (16) and identified an association of apathy with suicidality and poor quality of life (17, 18). Moreover, apathy has been reported to result in poor rehabilitation outcomes (19) and impaired functional capacity and recovery (20) in stroke survivors. Thus, apathy is a critical focus during stroke assessment and rehabilitation (21).

Apathy is a frequent and debilitating condition among SAH survivors, with a prevalence rate of 0%–68% (3, 22–26). Clinical factors potentially associated with post-SAH apathy include stroke severity (25), anterior communicating artery aneurysm (25), ventricular hematic density (25), decreased mental capacity (22), and impairments in the evaluation of the theory of mind and emotional recognition (26). Our group previously reported that in patients with ischemic stroke, apathy was associated with older age, stroke severity, physical function, global cognitive function, and depressive symptoms (16). However, the clinical course of apathy in patients with SAH remains unclear. We previously reported a chronic course of apathy in ischemic stroke: among patients with apathy at 3 months post-stroke, 51% and 42% still experienced persistent apathy at 9 and 15 months post-stroke, respectively (27). The predictors of persistent apathy in SAH survivors remain unclear, although longitudinal studies in stroke survivors have suggested that poor cognitive status, more comorbidities (28), and depressive symptoms (27, 29) predict a high risk of apathy. In addition, the impact of apathy on the clinical outcomes of patients with SAH remains to be determined. A previous study found that stroke patients with apathy scored lower on the Functional Independence Measurement (FIM) (30) upon discharge from an acute rehabilitation unit (19, 31). Conversely, a low level of apathy is associated with more favorable outcomes post-stroke (32), whereas a high level of apathy adversely affects physical function, participation, health perception, and physical health during the first 12 months after a stroke (28). A meta-analysis indicated that apathy negatively affects the global clinical outcomes of younger stroke survivors and those experiencing their first stroke (21). In addition, age, the World Federation of Neurosurgical Societies (WFNS) grade upon admission, intraventricular hemorrhage, and delayed cerebral ischemia have been identified as potential predictors of poor outcomes in patients with SAH (33–35).

Few high-quality trials have evaluated pharmacological and psychosocial treatments for apathy. Previous studies on pharmacological treatments have mainly focused on dopamine agonists (36) or stimulants, such as methylphenidate (37). Acetylcholinesterase (38), memantine (39), gingko biloba (11), agomelatine (40), and nefiracetam (41) may also effectively treat apathy (42). Nonpharmacological treatments for apathy include transcranial magnetic stimulation (43) and various occupational interventions, such as music and art therapy and psychomotor activities (44). In addition, a study suggested that strategy training (45), problem-solving therapy, and escitalopram may prevent apathy (46).

To the best of our knowledge, only 10 studies have specifically evaluated apathy in SAH (3, 9, 22–26, 47–49). None of these studies have examined the progression of the condition, predictors of its persistence, or its impact on functional outcomes. Furthermore, a major limitation of these studies is their small sample sizes; the smallest study included only 20 patients (50), and only 2 studies had more than 100 patients (*n* = 103) (3, 25). In addition, the durations of apathy assessments varied from ≤4 days (25) to 99 months (50). Moreover, 2 studies did not use a standardized method for assessing apathy (22, 23).

### Hypotheses

The proposed study will examine, for the first time, the 12-month course of apathy and its impact on functional outcomes in the largest cohort of SAH survivors to date. The first hypothesis is that 42% (51) of patients with apathy at baseline (3 month post-SAH, T1) will continue to exhibit apathy at 12 months after the first assessment. The second hypothesis is that poor cognitive status, a high level of comorbidity, and the presence of depressive symptoms at baseline (27–29) are predictors of persistent apathy. The third hypothesis is that patients with apathy have less favorable functional outcomes compared with their counterparts without apathy.

## Methods and analysis

### Participant recruitment

**Figure 1:**
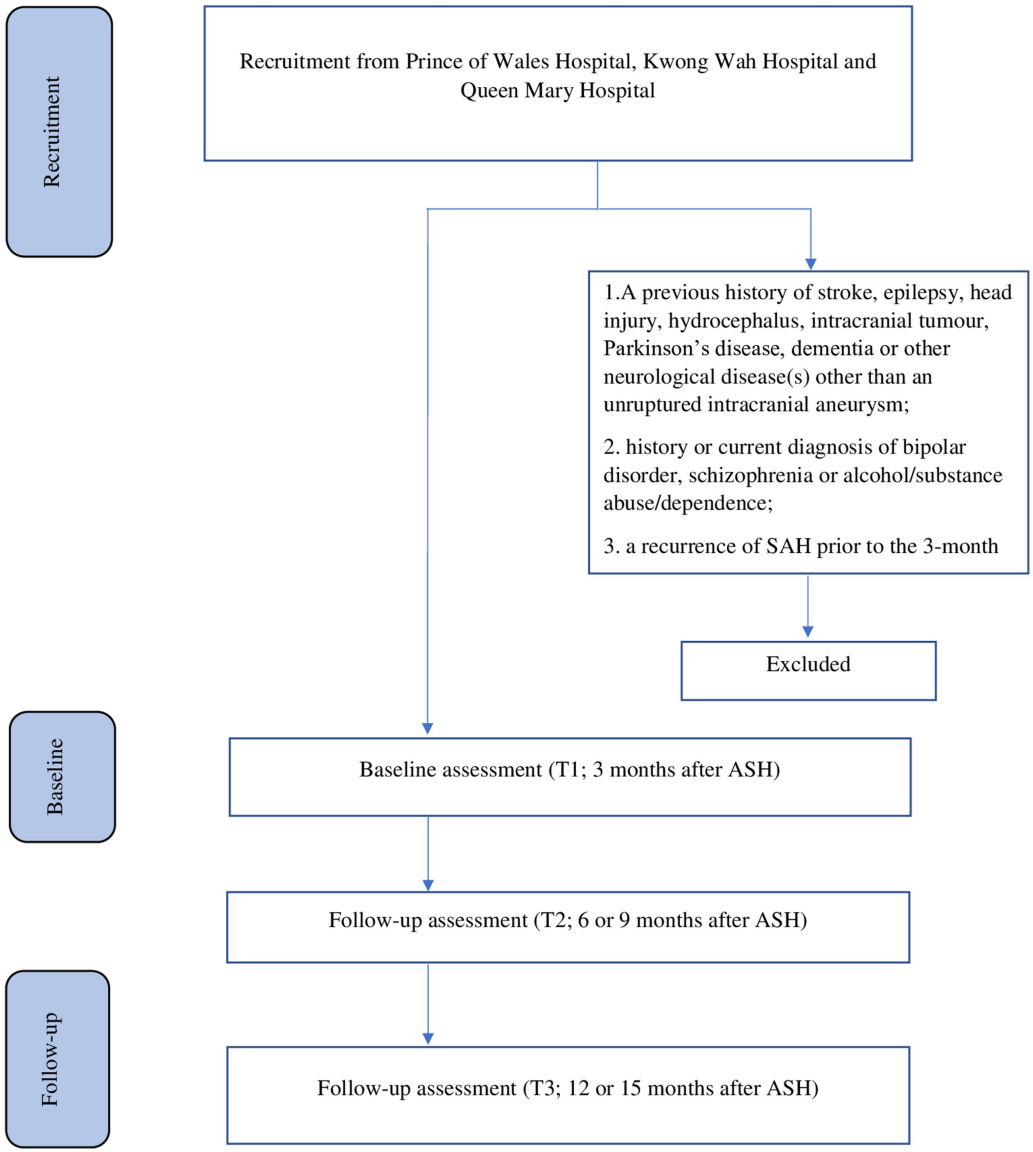
Details of recruitment.

The recruitment details are shown in Figure 1. Participants will be recruited from patients admitted with a first-ever SAH to the neurosurgical centers of three regional hospitals: Prince of Wales Hospital, Kwong Wah Hospital, and Queen Mary Hospital. All patients with SAH (*n* = 320, approximately 50–60 per center per year) who are consecutively admitted to these centers over a 24-month period will be invited to participate. A research assistant (RA) will visit the neurosurgical wards weekly to identify eligible patients and obtain written consent. We anticipate that 75% of these patients will meet our inclusion criteria and agree to participate (52), resulting in approximately 240 potential participants [320 × (75%)]. Assuming a dropout rate of 13% (53), we expect that 209 [240 × (100% − 13%)] patients will complete the 15-month follow-up assessment.

### Patient and public involvement statement

There will be no patient or public involvement.

### Eligibility criteria

#### Inclusion criteria

We will include participants aged ≥ 18 years with no upper age limit, of either male or female sex, and of Chinese ethnicity. Eligible participants must have experienced a spontaneous SAH resulting from an angiographically confirmed intracranial aneurysm within a maximum of 96 h before admission (3) and have the ability and willingness to provide informed consent.

#### Exclusion criteria

Exclusion criteria for this study include a previous history of stroke, epilepsy, head injury, hydrocephalus, intracranial tumor, Parkinson’s disease, dementia, aphasia, or other neurological diseases, with the exception of an unruptured intracranial aneurysm. In addition, individuals with a history or current diagnosis of bipolar disorder, schizophrenia, or alcohol/substance abuse/dependence and those who experience a recurrence of SAH prior to the 3-month assessment will be excluded from the study.

### Data collection

The proposed study is designed as a prospective cohort study. Written consent will be obtained from all patients or their next of kin. Demographic, psychosocial, and medical data will be collected from all patients. The number of and reasons for exclusions will be recorded. Details of the data collection schedule are provided in Table 1.

**Table 1:** Data collection schedule.

Study period	T1	T2	T3
Visit	**1**	**2**	**3**
Months	**3**	**6/9**	**12/15**
Informed consent	X		
Demographics, psychosocial and medical data.	X		
AES, FIM, IADL, BI, mRS, CCI, BDI, MMSE	X	X	X

Bold values of 1, 2, and 3 means times of visit; bold valued of 3, 6/9, and 12/15 means number of months after baseline.

### Neurological assessment

The neurological grade of patients at admission will be determined using the Glasgow Outcome Scale (54) and the WFNS grade scale. The WFNS scale categorizes patients from fully conscious with no motor or speech deficit (Grade I) to those with a Glasgow Coma Scale score of ≤6 (Grade V). Patients with a WFNS Grade I or II and Grade III or V will be classified as having good and poor grades, respectively.

For diagnostic assessments, all recruited patients will undergo delayed computed tomography (CT) scans of the brain 2 to 3 weeks after their initial presentation. Cerebral infarction due to delayed cerebral ischemia is defined as a new cerebral infarction on CT that cannot be attributed to any procedural causes (3). Clinical deterioration due to delayed cerebral ischemia is defined as presumably associated clinical deterioration that cannot be attributed to other potential causes (55). Hydrocephalus (acute or chronic) is characterized by persistent ventricular dilatation requiring the implantation of an internal shunt, such as a ventriculo-peritoneal shunt (3).

The severity of SAH on neuroimaging will be evaluated using the Fisher CT grade, which classifies findings into 4 levels: 1 for no blood visualized, 2 for diffuse or thin sheets (<1-mm thickness of vertical layers), 3 for localized clot and/or vertical layers (≥1-mm thickness), and 4 for the presence of intracerebral or intraventricular clots (56). In addition, details of the size of the ruptured aneurysm, its position (anterior cerebral artery, internal carotid/middle cerebral artery, and posterior circulation), the presence of intraventricular or intracerebral hemorrhage, and the treatment administered for the aneurysm will be recorded.

### Assessment of apathy

Apathy will be defined and quantified in accordance with the model proposed by Marin (57). An RA will operationally assess apathy using the Apathy Evaluation Scale (AES) (16, 58, 59), with an AES score of ≥37 indicating apathy (16). The study outcome will be the presence of apathy, categorized as either yes or no.

Three months after the onset of SAH (T1), patients and their caregivers will be assessed at a research clinic. This timing of the assessment is consistent with the schedule followed in other studies on apathy in patients with SAH (9) and stroke (16).

A trained RA will conduct interviews at a research clinic to assess apathy. The assessment will be based on interviews with the patients and their responses on the AES. This scale includes 18 items that evaluate the affective, behavioral, and cognitive domains of apathy by measuring motivational variables such as productivity, initiative, effort, emotional responsivity, novelty seeking or curiosity, perseverance, and social engagement. Responses are scored on a 4-point Likert-type scale, ranging from “not at all true/characteristic” to “very much true/characteristic,” with higher scores indicating more severe apathy. The AES has good reliability (58) and validity (60), and the internal consistency coefficient, test–retest reliability, and inter-rater reliability of the Chinese version of this scale are 0.90, 0.90, and 0.86, respectively (59). The AES was originally validated in a cohort with mixed diagnoses, namely, stroke, dementia, and depression (57), and has been widely used in various populations (60). To the best of our knowledge, no disease-specific measure of apathy has been developed for SAH or stroke. However, the AES has been used to assess apathy in patients with SAH (9, 24–26), and our group successfully used this scale to assess apathy in stroke (16).

### Assessment of functioning

A trained RA blinded to the patients’ apathy data will assess each patient’s level of functioning using the FIM (61), Lawton Instrumental Activities of Daily Living Scale (IADL) (62), Barthel Index (BI) (63), and modified Rankin Scale (mRS) (64). These measures have been previously used to measure functional outcomes after SAH (52, 65, 66). The FIM includes 18 items across 6 subscales: self-care, sphincters, mobility, communication, psychosocial, and cognition. Each item is rated on a 7-point scale from 1 (patient requires total assistance) to 7 (patient is completely independent). The overall score ranges from 18 to 126 points. The FIM is considered valid with good inter-rater agreement (67). The IADL scale is used to assess independent living skills, such as using a telephone, shopping, preparing food, performing housekeeping and laundry, transportation, managing medication responsibly, and handling finances. An IADL score of 18 is considered an excellent outcome. This scale has good content validity and excellent inter-rater reliability (0.99), test–retest reliability (0.90), and internal consistency (0.86) (62). The BI is used to measure independence based on 10 tasks, scored according to the amount of time or assistance required by the patient. The total BI score ranges from 0 to 20, with lower scores indicating greater dependency. The BI has favorable construct validity and predictive validity and moderate inter-and intra-reliability (0.4–0.6) (68). The mRS was previously used to rank outcomes (e.g., death, disability, and dependence) after stroke or SAH (53). The total mRS score ranges from 0 (no symptoms) to 6 (death), with 0 indicating an excellent outcome. In addition, a caregiver will complete a checklist on the patient’s post-SAH changes to assist with scoring, as recommended in a previous study (69). The construct validity and inter-rater reliability (0.6) of this checklist are excellent and moderate, respectively (68).

### Other assessments

A trained RA will evaluate the levels of comorbidity, global cognitive functioning, and depressive symptoms using the Charlson Comorbidity Index (CCI) (70), Chinese version of the Beck Depression Inventory (BDI) (71), and Hong Kong version of the Montreal Cognitive Assessment (MoCA) (72), respectively. The CCI is the most widely used comorbidity adjustment method. It involves weighting 19 diseases from 1 to 6 points, and the sum of the weights is adjusted to produce final scores ranging from 0 to 33. This index has good validity and test–retest reliability and moderate-to-good inter-rater reliability (73). The BDI, consisting of 21 items, is scored on a 4-point Likert scale, with total possible scores ranging from 0 to 63. Higher scores indicate more severe depressive symptoms; specifically, scores of 0–9, 10–18, 19–30 and > 30 indicate no/minimal, minor/moderate, moderate/severe, and severe symptoms, respectively. The BDI has good internal consistency (0.86) (71), retest reliability (0.73–0.96), and validity (74). The MoCA is a one-page test with a maximum score of 30. It is used to evaluate seven cognitive domains, namely, visuospatial/executive functions, naming, verbal memory registration and learning, attention, abstraction, 5-min delayed verbal memory, and orientation. This scale has excellent discriminant validity, internal consistency (0.77), test–retest reliability (0.87), and inter-rater reliability (0.99) (72, 75). Any physiotherapy or cognitive/neuropsychological rehabilitation received by the patients will also be recorded.

All patients will undergo follow-up assessments of apathy and functioning at 9 (T2) and 15 months (T3) post-SAH or at 6 and 12 months after the first assessment. The AES, FIM, IADL, BI, mRS, MoCA, and BDI will be re-administered during the follow-up assessments.

### Sample size estimation

Two hundred and forty patients will be recruited. Based on an estimated apathy frequency of 42% (7, 26), we expect to identify 101 (240 × 42%) cases of apathy. A sample size of 101 and 139 (i.e., 240–101) patients with and without apathy, respectively, would provide a power of 80% for the detection of any differences in functional outcomes between these 2 groups, assuming an effect size of apathy of ≥0.4. In a previous study, the effect size of apathy on functional outcomes in stroke survivors (Cohen’s d) was 2.4 (19).

Assuming a dropout rate of 13% (53), we anticipate that 88 (101 × 87%) patients with apathy will complete the follow-up visits. Although the rate of persistent apathy after SAH is unknown, previous studies on general stroke patients have reported persistent apathy rates ranging from 42 to 51% (27). Accordingly, our sample size is expected to achieve a power of 80% for the identification of predictors of persistent apathy with an odds ratio of ≥2.0. This is below the odds ratio of 3.3 that we previously reported for the impact of depressive symptoms on the 1-year course of apathy in stroke survivors (27). We will assume that the R^2^ value of the other variables in the multivariate logistic regression (76) will be 0.21 (51).

### Statistical analyses

All variables will be tested for normality using the Kolmogorov–Smirnov test, with a significance threshold of *p* < 0.05. The number and percentage of patients with persistent apathy will be calculated. To identify the predictors of persistent apathy, the baseline MoCA, BDI, and CCI scores as well as other demographic, clinical, and CT scan characteristics and physiotherapy or cognitive/neuropsychological rehabilitation received will be compared between patients with and without apathy at T2 using the χ2 test, Student’s t test, or Mann–Whitney U test as appropriate. A stepwise logistic regression will then be performed to assess the importance of the baseline MoCA, BDI and CCI scores, together with other significant variables identified in the univariate analyses. The same analyses will be repeated using data obtained at T3. We will also test a series of generalized estimating equation models to evaluate the associations of the baseline demographic, clinical, and CT scan characteristics with the risk of apathy across all follow-up assessments (T1/T2/T3). First, we will generate a univariate model to fit a logistic regression. Next, we will examine associations between demographic variables and the risk of apathy. The second model will include CT scan characteristics, whereas the final model will include the baseline MoCA, BDI, and CCI scores. The level of significance will be set at 0.05. We will perform two sub-analyses. First, the analysis will be repeated by substituting the MoCA total score with the executive functioning index score of the MoCA (77). Second, the analysis will be repeated for three subdomains (i.e., cognitive, affective, and behavioral) of the AES (78).

To examine the impact of apathy on functional outcomes, the FIM, IADL, BI, and mRS scores of the groups with and without apathy at T1 will be compared using a Student’s t test. The aforementioned outcome scores will then be adjusted for other putative outcome predictors (age, WFNS grade upon admission, intraventricular hemorrhage, and delayed cerebral ischemia) using analysis of covariance. Correlation of the functional outcome scores with the presence of apathy and other putative predictors will be computed using Pearson’s or Spearman’s correlation coefficients, as appropriate. Finally, the presence of apathy and significant predictors identified in the univariate analysis will be entered into a regression analysis of the functional outcome scores. This analysis will be repeated for T2 and T3.

## Ethics and dissemination

This study will prioritize ethical considerations by seeking informed consent from all participants and ensuring their privacy and confidentiality. Participant safety will be closely monitored throughout the study, and measures will be implemented to minimize any potential risks. The study has been approved by the relevant institutional review board or ethics committee and adheres to established guidelines and regulations governing human research.

The study findings will be shared through peer-reviewed journal publications, national and international conferences, and social media platforms. This multi-faceted approach will maximize the impact of the research by reaching the scientific community, relevant stakeholders, and the wider public. These dissemination efforts will provide valuable information for informed decision-making, promote apathy evaluation, and facilitate the development of prevention strategies, rehabilitation programs, and therapeutic options.

## Risks and contingency plans

The main risks of study are the slow recruitment of patients and loss to follow-up. The corresponding mitigation plans include inviting more regional hospitals to join the study and conducting home visits to perform follow-up assessments for those who are unable or unwilling to visit the research clinics.

## Discussion

We will attempt to recruit a homologous patient population by narrowing the ethnicity and duration of SAH criteria. Patients with other potential causes of apathy, including psychiatric disorders, alcohol/substance abuse, and neurological disorders, will be excluded. Various predictors of the risk of apathy will be included, such as measures of mood and cognitive function. A conservative estimate of effect size will be used to ensure the inclusion of an adequate sample size.

This will be the first large-scale 1-year follow-up study of apathy in SAH survivors. The findings of this study will provide valuable data to advance our understanding of the clinical course of apathy in this population. The findings will have clinical relevance by providing essential information to patients, caregivers, and clinicians; promoting the evaluation of apathy; and facilitating the development of prevention strategies, rehabilitation programs, and therapeutic options.

## Ethics statement

The studies involving humans were approved by Joint Chinese University of Hong Kong-New Territories East Cluster Clinical Research Ethics Committee (CREC Ref. No.: 2023.339). The studies were conducted in accordance with the local legislation and institutional requirements. Written informed consent for participation was not required from the participants or the participants’ legal guardians/next of kin because it is a study protocol and recruitment of participants have not started.

## Author contributions

WT: Conceptualization, Data curation, Formal analysis, Methodology, Writing – original draft, Writing – review & editing. KW: Conceptualization, Data curation, Formal analysis, Methodology, Writing – original draft, Writing – review & editing.
